# Genetic predisposition to type 2 diabetes mellitus and aortic dissection: a Mendelian randomisation study

**DOI:** 10.3389/fcvm.2024.1382702

**Published:** 2024-07-22

**Authors:** Yaodong Sun, Dongdong Du, Jiantao Zhang, Linlin Zhao, Bufan Zhang, Yi Zhang, Tianxu Song, Naishi Wu

**Affiliations:** ^1^ Department of Cardiovascular Surgery, Tianjin Medical University General Hospital, Tianjin, China; ^2^ Department of Cardiovascular Surgery, Shandong Provincial Hospital Affiliated to Shandong First Medical University, Jinan, Shandong, China; ^3^ Shanxi Bethune Hospital, Shanxi Academy of Medical Sciences, Tongji Shanxi Hospital, Third Hospital of Shanxi Medical University, Taiyuan, China; ^4^ Tongji Hospital, Tongji Medical College, Huazhong University of Science and Technology, Wuhan, China

**Keywords:** diabetes, aortic dissection, Mendelian randomisation, genome-wide association study, epidemiology

## Abstract

**Background:**

This Mendelian randomization (MR) study aimed to explore the causal relationship between the genetic predisposition to type 2 diabetes mellitus (T2DM) and aortic dissection (AD), and to assess associations with genetically predicted glycemic traits. The study sought to verify the inverse relationship between T2DM and AD using a more robust and unbiased method, building on the observational studies previously established.

**Materials and methods:**

The study employed a two-sample and multivariable MR approach to analyze genetic data from the DIAbetes Meta-ANalysis of Trans-Ethnic association studies (DIAMANTE) with 74,124 cases and 824,006 controls, and the Meta-Analyses of Glucose and Insulin-Related Traits Consortium (MAGIC) involving up to 196,991 individuals. For AD data, FinnGen Release 10 was used, including 967 cases and 381,977 controls. The research focused on three foundational MR assumptions and controlled for confounders like hypertension. Genetic instruments were selected for their genome-wide significance, and multiple MR methods and sensitivity analyses were conducted.

**Results:**

The study revealed no significant effect of genetic predisposition to T2DM on the risk of AD. Even after adjusting for potential confounders, the results were consistent, indicating no causal relationship. Additionally, glycemic traits such as fasting glucose, fasting insulin, and HbA1c levels did not show a significant impact on AD susceptibility. The findings remained stable across various MR models and sensitivity analyses. In contrast, genetic liability to T2DM and glycemic traits showed a significant association with coronary artery disease (CAD), aligning with the established understanding.

**Conclusion:**

Contrary to previous observational studies, this study concludes that genetic predisposition to T2DM does not confer protection against AD. These findings underscore the imperative for further research, particularly in exploring the preventative potential of T2DM treatments against AD and to facilitate the development of novel therapeutic interventions.

## 1 Introduction

Aortic dissection (AD) stands as the most frequently encountered catastrophic event afflicting the aorta (1). Hospital records of acute aortic syndromes suggest an incidence rate of 3–5 per 100,000 individuals annually, with a spike to 35 per 100,000 in those aged 65–75 (2, 3). AD also imposes a significant economic burden on both the patients’ families and society (4). At present, no pharmacological intervention has been demonstrated to be effective in the prevention of AD.

Controversy surrounds the influence of diabetic conditions on AD. Hypertension is typically difficult to control in diabetic patients, which leads to weakness of the medial layer of the aorta and promotes AD. However, a paradox emerges as studies reveal a reduced prevalence of AD in patients with type 2 diabetes mellitus (T2DM) (5, 6). Meta-analyses corroborate this observation, showcasing a lower AD incidence among T2DM patients (7, 8). Observational studies often struggle to establish causality due to the presence of potential confounders and biases. This is in contrast to randomized controlled trials. As a result, the findings and interpretations of the relationship between T2DM and AD in observational studies may be skewed. Moreover, the exact mechanisms underlying this inverse relationship remain unclear. Current hypotheses fluctuate between the protective role of glycated crosslinks in aortic tissue and the beneficial effects of certain T2DM treatments (9, 10). The pivotal question of T2DM's intrinsic protective propensity against AD remains unresolved.

Mendelian randomization (MR) analysis is an epidemiological design that can strengthen causal inference by using genetic variants as instrumental variables (IVs) for an exposure (11). The MR design, characterized by its immunity to confounding biases and reverse causality, owes its robustness to the random assortment of genetic alleles at conception and their insusceptibility to modification by diseases (12). Previous MR studies have shown that genetic predisposition to T2DM did not play a causal role in abdominal aortic aneurysm (AAA) but decreases the risk of thoracic aortic aneurysm (TAA) (13, 14). Our MR study aims to delineate the causal relationship between T2DM genetic predisposition and AD while examining associations with genetically predicted glycemic traits as supplementary analyses.

## 2 Methods

### 2.1 Study design

**Figure 1:**
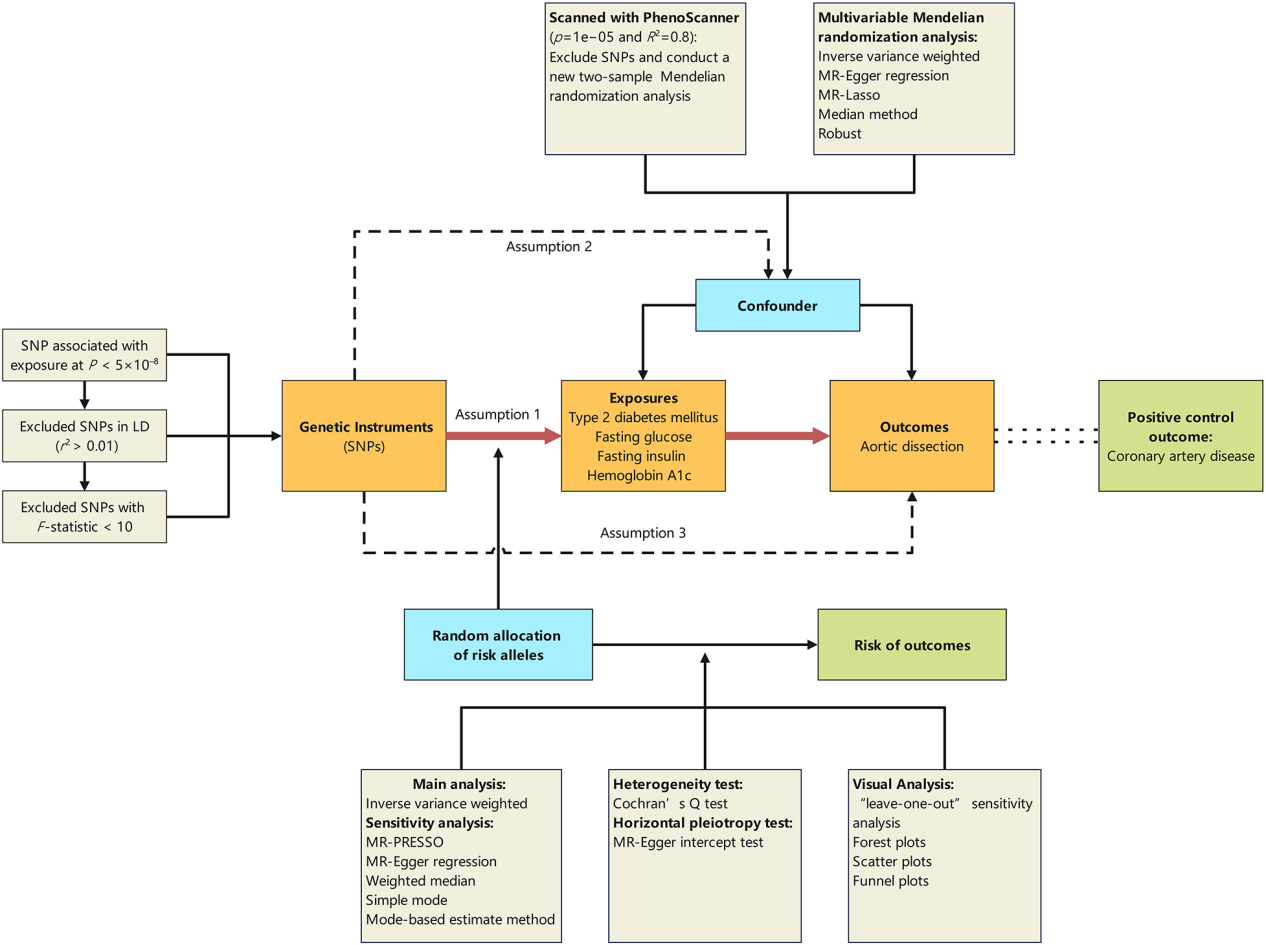
Schematic representation of the Mendelian randomisation design. The traditional assumptions of Mendelian randomisation are that the genetic instrumental variable is associated with the risk factor (assumption 1); the variants should not be associated with confounders (assumption 2); and that the variants should influence the outcome only through effects on the risk factor under investigation (assumption 3). SNP, single nucleotide polymorphism; LD, linkage disequilibrium; *p*, probability value; *r*^2^, coefficient of determination.

This two-sample and multivariable MR study was designed to explore the causal effect of T2DM and glycemic traits on the risk of AD. The ethics committee at each institutional review board review board authorized all participants’ written informed consent in separate studies. Further ethical approval or consent was deemed unnecessary. Our MR study was predicated on three foundational assumptions: (1) the IVs are related to exposures of interest; (2) the IVs are not related to the confounders of the exposure-outcome relation; (3) the IVs’ links with the outcome are only via the exposure of interest (15). To eliminate potential confounding factors, specifically the impact of hypertension, two types of secondary analyses were conducted. To address potential confounders, particularly hypertension's influence, we conducted two secondary analyses. First, a two-sample MR study was used to exclude Single Nucleotide Polymorphisms (SNPs) linked to hypertension. Then, drawing upon epidemiological information on T2DM and AD, we conducted two-sample MR analysis to explore factors that may influence the onset of these two diseases. In this context, we examined factors such as blood pressure, lipids, diet, sleep, physical activity, smoking, alcohol, socioeconomic elements, and stress. Factors related to both T2DM and AD were deemed confounders and included in further multivariate MR studies to assess T2DM's independent role. We used coronary artery disease (CAD) as a positive control to ensure IVs showed expected associations with a common disease, reducing selection bias risk (16). Additionally, the UK Biobank's AD database served as a validation set. Subsequent sections detail the data sources, genetic instrument selection, and statistical analyses (Figure 1; Table 1; Supplementary Table S1).

**Table 1:** Information of included studies and consortia.

Exposure/Outcome	Traits	Consortium/First author	Participants	Web source
Exposures	Type 2 diabetes	DIAbetes Meta-Analysis of Trans-Ethnic association studies (DIAGRAM)	74,124 cases and 824,006 controls	https://diagram-consortium.org/downloads.html
	Fasting glucose (mmol/L)	Meta-Analyses of Glucose and Insulinrelated traits Consortium (MAGIC)	196,991 individuals	https://magicinvestigators.org/downloads/
	Fasting insulin (pmol/L)	Meta-Analyses of Glucose and Insulinrelated traits Consortium (MAGIC)	196,991 individuals	https://magicinvestigators.org/downloads/
	Hemoglobin A1c (%)	Meta-Analyses of Glucose and Insulinrelated traits Consortium (MAGIC)	196,991 individuals	https://magicinvestigators.org/downloads/
Outcomes	Aortic dissection	FinnGen	967 cases and 381,977 controls	https://www.finngen.fi/fi
	Aortic dissection	UK biobank	127 cases	https://pan.ukbb.broadinstitute.org/
	Coronary artery disease	CARDIoGRAMplusC4D (Coronary ARtery DIsease Genome wide Replication and Meta-analysis (CARDIoGRAM) plus The Coronary Artery Disease (C4D) Genetics) and UK biobank	63,731 cases and 276,868 controls	http://www.cardiogramplusc4d.org/data-downloads/

### 2.2 Exposure

We sourced genetic data for T2DM from the DIAbetes Meta-ANalysis of Trans-Ethnic association studies (DIAMANTE) consortium's GWAS meta-analysis, encompassing 74,124 cases and 824,006 controls, all of European ancestry (17). T2DM in the original GWAS was defined by diagnostic fasting glucose (FG), fasting insulin (FI), 2 h plasma glucose or hemoglobin A_1c_ (HbA_1c_) levels; use of glucose-lowering medication (by Anatomical Therapeutic Chemical code or self-report); or T2DM history from electronic medical records, self-report and varying combinations of each, depending on the contributing cohort.

For glycemic traits like FI, FG, and HbA_1c_, we referred to the Meta-Analyses of Glucose and Insulin-Related Traits Consortium (MAGIC) data, which includes information from up to 196,991 European participants.

To confirm potential confounding factors, we included variables such as Systolic Blood Pressure (SBP), Diastolic Blood Pressure (DBP), Pulse Pressure (PP), High-Density Lipoprotein cholesterol (HDL-C) (18), Low-Density Lipoprotein cholesterol (LDL-C) (18), Total Cholesterol (TC) (18), Triglycerides (TG) (18), dietary intakes (protein, fat, sugar, carbohydrates) (19), socioeconomic factors [educational level (20), annual household income, Townsend Deprivation Index (TDI)], lifestyle factors (skipping breakfast (21), napping (22), daytime sleepiness (23), sleep duration (24), insomnia (25), leisure screen time (26), moderate-to-vigorous intensity physical activity during leisure time (MVPA) (26), sedentary behavior at work (26), environmental stress and adversity (27), smoking (smoking initiation, daily smoking, smoking cessation, smoking initiation) (28), drinking (weekly alcohol consumption data (28), alcohol abuse (29). Detailed database sources for each variable are provided in Supplementary Table S2.

We included SNPs correlating with the selected traits at a genome-wide significance threshold (*p *< 5 × 10^−8^). We ensured the avoidance of weak instrument bias by prioritizing SNPs with *F*-statistics exceeding 10 (30). The absence of rsid in the DIAMANTE's T2DM database necessitated its determination via SNP-nexus (https://www.snp-nexus.org/v4/) and the National Library of Medicine (https://www.ncbi.nlm.nih.gov/snp/?term=), reliant on chromosome and position data. In the presence of linkage disequilibrium (LD) (*r*^2 ^> 0.001), preference was given to the SNP boasting the strongest association, thereby safeguarding the independence assumption. This yielded 150 SNPs for T2DM, 38 for FI, 70 for FG, and 75 for HbA1c collectively termed as primitive groups. The proportion of variance in the exposure factors explained by the instrumental variables for T2DM, FG, FI, and HbA1c are 7.32%, 4.32%, 1.42%, and 5.76%, respectively (Supplementary Table S1).

Each SNP was scanned with PhenoScanner to identify and mitigate potential pleiotropic effects. SNPs correlating with established risk factors of AD, including hypertension within the European population, were filtered out utilizing a threshold of *p *= 1 × 10^−5^ and *R*^2 ^= 0.8. Consequently, 17 SNPs of T2DM, 7 SNPs of FI, 11 SNPs of FG, and 6 SNPs of HbA_1c_ were excluded (Supplementary Table S3). The remaining SNPs constituted the adjustment groups.

### 2.3 Outcome selection

GWAS summary statistics for AD were obtained from FinnGen Release 10, a project mapping genotype-phenotype correlations using Finnish biobank data. This included genome and health data from 1,069 AD individuals and 377,277 controls, characterized by 473,681 genotyped SNPs. Aortic dissection diagnosis was based on hospital records, using ICD-10 codes I71.00, I71.01, I71.09, and ICD-9 code 4410. Endpoint and control definitions are available at https://www.finngen.fi/en/researchers/clinical-endpoints. The UK Biobank database, with 127 cases, served as a validation set (http://www.nealelab.is/uk-biobank).

The genetic data for coronary artery disease were obtained from a meta-analysis of UK Biobank SOFT CAD GWAS (interim release) with CARDIoGRAMplusC4D (Coronary ARtery DIsease Genome wide Replication and Meta-analysis (CARDIoGRAM) plus The Coronary Artery Disease (C4D) Genetics) 1,000 Genomes-based GWAS and the Myocardial Infarction Genetics and CARDIoGRAM Exome (31).

### 2.4 Statistical analysis

Statistical significance was set at a two-sided 0.05 level, if the *p*-value is less than the Bonferroni-corrected threshold of 1.11 × 10^−3^, we consider it indicative of a strong correlation. The selection of instrumental variables (IVs) is detailed in the “Exposure” section. For the primary analysis, we combine Wald ratio together in fixed effect meta-analysis, where the weight of each ratio is the inverse of the variance of the SNP-outcome association. The IVW method provides the most precise and robust estimates when three pivotal assumptions regarding instrumental variables are satisfied (32). MR-Presso, MR-Egger, weighted median, simple mode, and Mode based estimate method were used for sensitivity analysis. Heterogeneity in our analysis was assessed using the Cochran *Q* test, with a *p*-value of less than 0.05 deemed significant. A *Q* value substantially exceeding its degrees of freedom indicates evidence of heterogeneity and suggests the presence of invalid instruments (33). MR-Egger regression was utilized to analyze potential unbalanced horizontal pleiotropy, combining the Wald ratio into a meta-regression with an intercept and slope parameter. This approach estimates the causal effect while adjusting for any directional pleiotropy (34). Leave-one-out analyses were used to verify result reliability. Multivariable MR analyses also utilized five methods (IVW, MR-Egger, MR-Lasso, median, and robust), which used to learn about the causal effect of two or more exposures on an outcome (35, 36). Statistical analyses were performed using R software (version 4.3.1) and packages including TwoSampleMR (37), vroom, MVMR (38), MRPRESSO (39), and others.

## 3 Results

### 3.1 T2DM and AD

**Figure 2:**
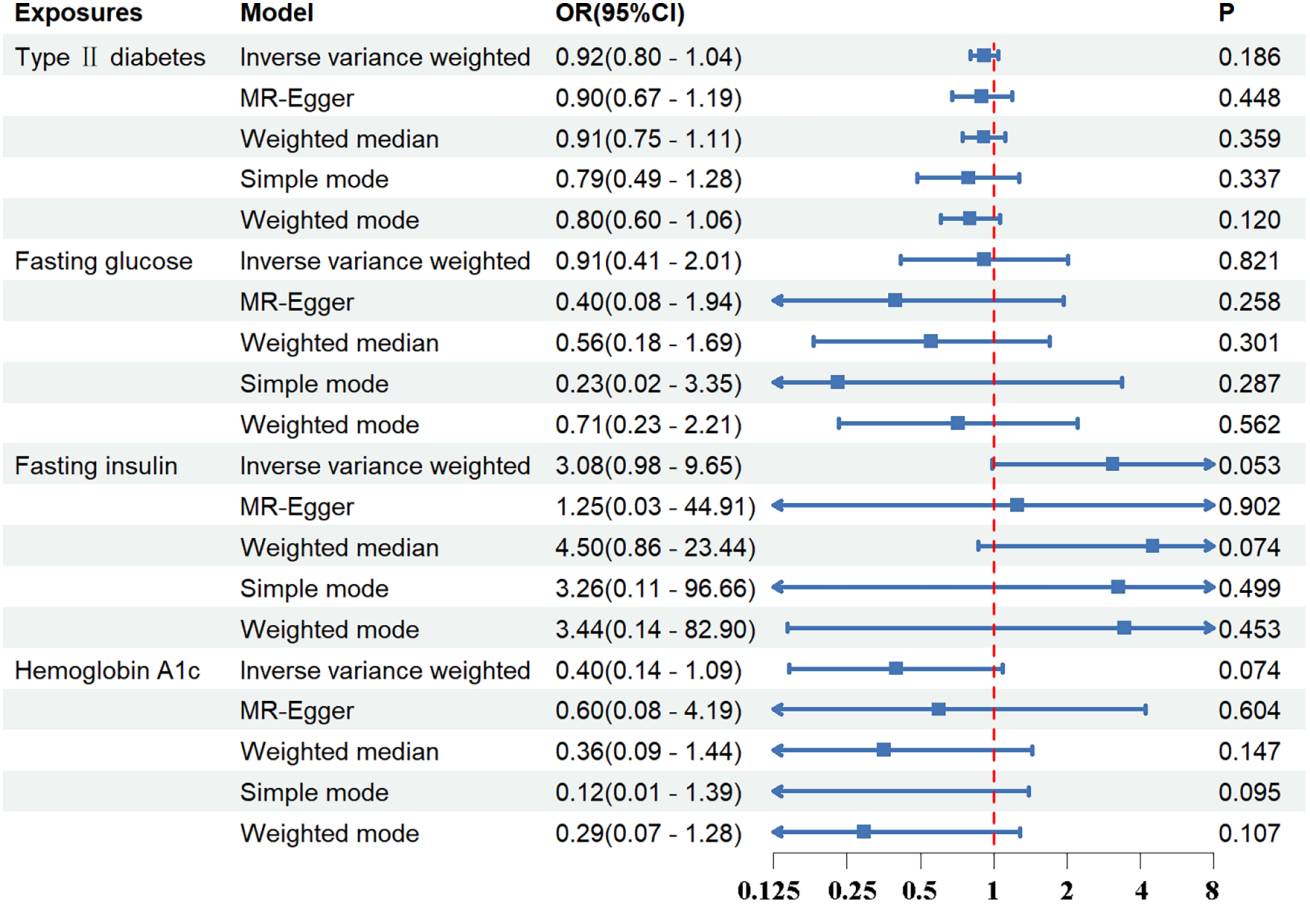
The association between exposures and aortic dissection using the inverse variance weighted, MR-egger, weighted median, simple mode, weighted mode method. OR, odds ratio; CI, confidence interval; *p*, probability value.

**Figure 3:**
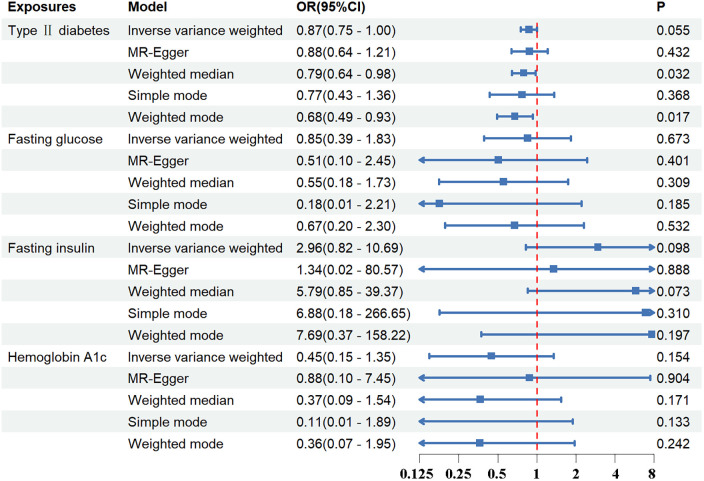
The association between exposures adjustment groups and aortic dissection using the inverse variance weighted, MR-egger, weighted median, simple mode, weighted mode method. OR, odds ratio; CI, confidence interval; *p*, probability value.

Standard IVW analysis showed no convincing effect of genetic predisposition to T2DM on AD, with an odds ratio (OR) of 0.92 [95% confidence interval (CI), 0.80–1.04; *p *= 0.186]. The MR-Egger and weighted median models, which are more robust to directional pleiotropy, showed similar findings (MR-Egger OR = 0.90; 95%CI, 0.67–1.19; *p *= 0.448; weighted median OR = 0.91, 95%CI, 0.75–1.11; *p *= 0.359) compared with the IVW model. This consistency in outcomes was maintained even in the adjustment group (IVW OR = 0.87; 95%CI, 0.75–1.00; *p *= 0.055). The outcomes in the UK Biobank database were similarly concluded (IVW *p *= 0.290; MR-Egger *p *= 0.295; weighted median *p *= 0.412). The results are tabulated for further review (Figures 2, 3; Supplementary Tables S4, S7).

Through Cochran *Q* test, it was found that there was no significant heterogeneity among SNPs (Supplementary Table S5). Through MR-Egger regression analysis, it is confirmed that there is no significant horizontal pleiotropy in the above analysis data (Supplementary Table S6). A comprehensive sensitivity analysis, facilitated by the leave-one-out method, affirmed the stability of the results, indicating that the exclusion of any individual SNP would not materially influence the overall conclusion (Supplementary Figure S2). For an enhanced visual representation and interpretation of the data, scatter and funnel plots have been provided (Supplementary Figure S1), and the forest plots were presented in Supplementary Figure S2.

### 3.2 Glycemic traits and AD

Regarding FG, FI, and HbA_1c_ levels, the standard IVW analysis did not reveal a significant impact on AD susceptibility (FG: OR = 0.91; 95%CI, 0.41–2.01; *p *= 0.821; FI: OR = 3.08; 95%CI, 0.98–9.65; *p *= 0.053; HbA_1c_: OR = 0.40; 95%CI, 0.14–1.09; *p *= 0.074) (Figures 2, 3; Supplementary Tables S4, S7).

The Cochran *Q* test revealed significant heterogeneity within HbA_1c_ and the primitive group of FG, while other SNP groups displayed no notable heterogeneity (Supplementary Table S5). Additionally, the data analysis did not indicate significant horizontal pleiotropy (Supplementary Table S6). Sensitivity analysis, conducted via the leave-one-out method, suggested the overall conclusion remained stable when any given SNP was omitted from the study (Supplementary Figures S4, S6, S8). Comprehensive visualizations of the study's findings, including scatter plots and funnel plots, are depicted in Supplementary Figures S3, S5, S7, while detailed forest plots are illustrated in Supplementary Figures S4, S6, S8.

### 3.3 Confounding factors and multivariate MR analysis

**Figure 4:**
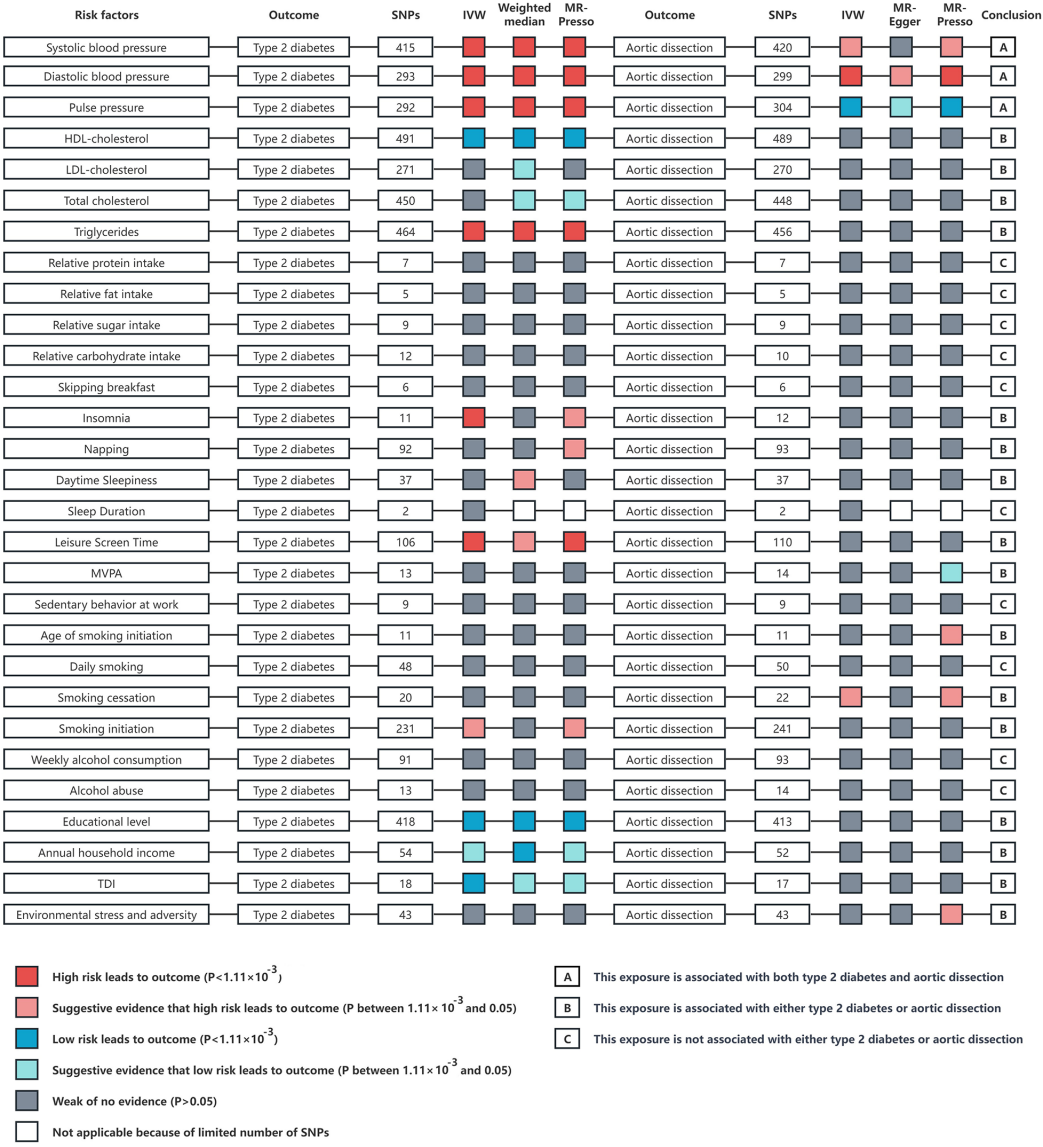
An overview of the Mendelian randomization study results on risk factors such as blood pressure, blood lipids, diet, sleep, physical activity, smoking, alcohol consumption, socioeconomic factors, and environmental stress in relation to type 2 diabetes and aortic dissection. The analysis methods include inverse variance weighted, weighted median, and MR-PRESSO analysis. IVW, inverse variance weighted; *p*, probability value; HDL, high density lipoprotein; LDL, low density lipoprotein; MVPA, moderate to vigorous intensity physical activity during leisure time; TDI, townsend deprivation index.

A comprehensive two-sample MR study was conducted to identify confounding factors and it was found that SBP, DBP, PP, TG, insomnia, napping, daytime sleepiness, leisure screen time, and smoking initiation were positively correlated with T2DM. Conversely, HDL-C, LDL-C, TC, educational level, annual household income, and the TDI were negatively correlated with T2DM. SBP, DBP, age of smoking initiation, smoking cessation, and environmental stress and adversity were positively correlated with AD, whereas PP and MVPA were negatively correlated with AD (Figure 4; Supplementary Tables S4, S8).

In light of the aforementioned findings, we conducted multivariate MR analysis using SBP, DBP, and PP as confounding factors alongside the exposure variables. Considering that PP is essentially determined by both SBP and DBP, we repeated the multivariate MR analysis using only SBP and DBP as confounding factors with the exposure variables. This approach was adopted to prevent the introduction of bias.

**Figure 5:**
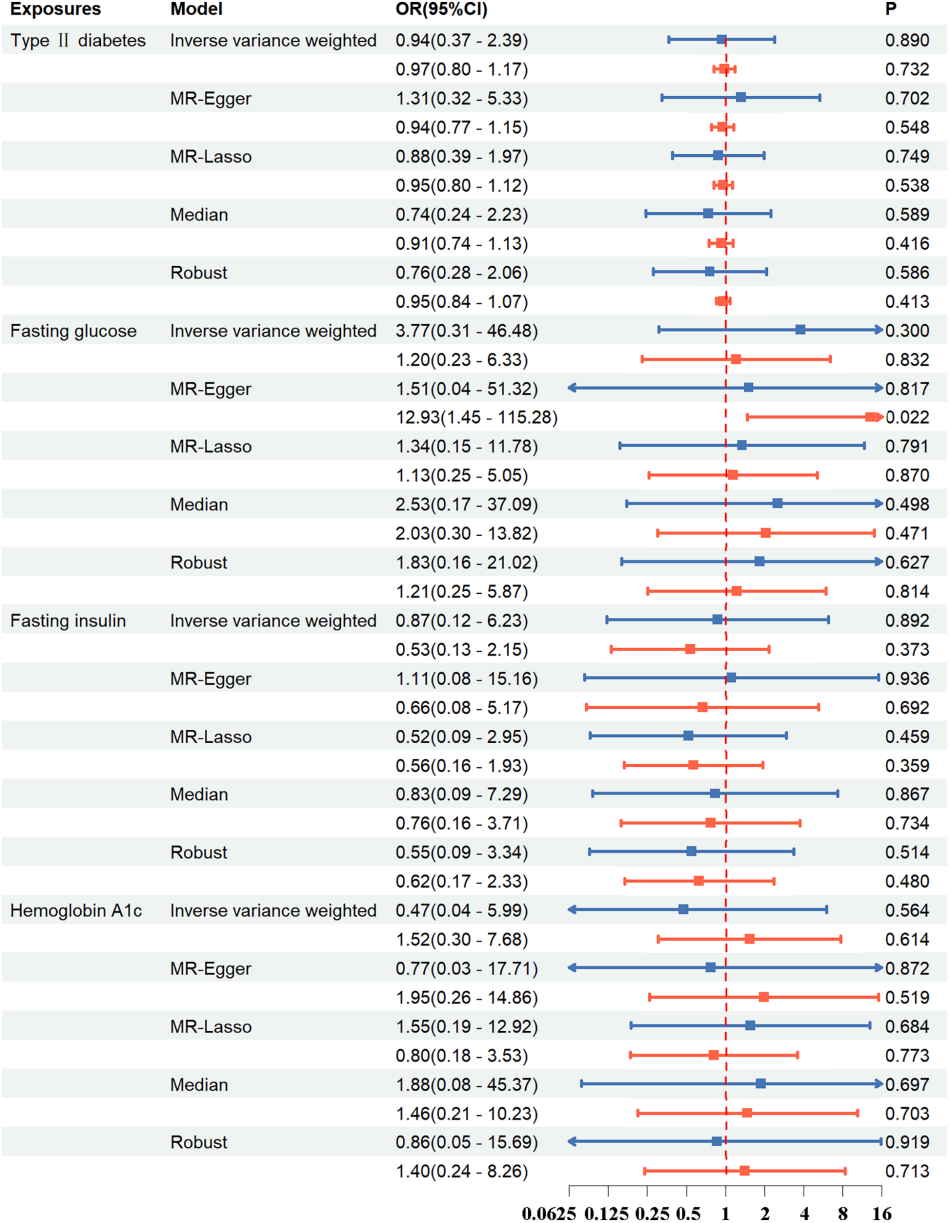
In the multivariable MR study, the association between exposure and aortic dissection was investigated using methods such as inverse variance weighted, MR-egger, MR-lasso, median, and robust. Blue line: Exposures include type 2 diabetes, systolic blood pressure, diastolic blood pressure, pulse pressure; Red line: Exposures include type 2 diabetes, systolic blood pressure, diastolic blood pressure. OR, odds ratio; CI, confidence interval; *p*, probability value.

The results demonstrated that T2DM, FG, FI, and HbA1c did not exhibit a significant correlation with AD in both instances of the multivariate MR studies (Figure 5; Supplementary Table S8).

### 3.4 T2DM, glycemic traitsand and CAD

The robustness of the IVs was further assessed against CAD to confirm whether it yielded significant positive associations as previously established. Genetic liability to T2DM and glycemic traits was significantly associated with CAD under IVW models (T2DM: OR = 1.12; 95%CI, 1.08–1.16; *p *< 0.001; FG: OR = 1.26; 95%CI, 1.04–1.53; *p *= 0.018; FI: OR = 1.91; 95%CI, 1.31–2.79; *p *< 0.001; HbA_1c_: OR = 1.44; 95%CI, 1.09–1.90; *p *= 0.010) (Supplementary Table S4). Comprehensive visualizations of the study's findings, including scatter plots and funnel plots, are depicted in Supplementary Figures S9, S10.

## 4 Discussion

Our MR study suggests that there is no significant association between the genetic predisposition to T2DM and AD. Even after adjusting for potential confounders, such as hypertension, the results were steadfast, underscoring the robustness of our conclusions. In the realm of FG, FI, and HbA_1c_ levels, our study did not identify any causal relationships with AD incidence. These findings present a stark contrast to previous observational studies, which have consistently indicated a substantial negative correlation between T2DM and AD.

T2DM, known for increasing the risk of peripheral, coronary, and cerebrovascular diseases, has paradoxically been linked with a lower risk of aortic aneurysm and dissection. The first evidence of this inverse relationship appeared in 1997, showing fewer cases of AAA in diabetic individuals (40). Despite differences between thoracic and abdominal aortic diseases, this finding sparked interest in the diabetes-aortic disease link. Prakash's analysis of the Nationwide Inpatient Sample (NIS) data confirmed diabetes's association with reduced AD hospitalizations (41), a conclusion echoed by recent studies (5, 6, 42–44). However, these studies often rely on self-reported T2DM data and focus predominantly on hospitalized patients, which may introduce selection bias and obscure the true prevalence of T2DM in AD patients. The inherent limitations in observational studies, like residual confounders, challenge establishing a clear causal link between these diseases. This highlights the value of MR studies in delineating the causal relationships between T2DM and aortic diseases.

Aortic disease is distinctly divided at the ligamentum arteriosum. Above this, the aorta in aneurysm disease typically shows a smooth, non-calcified surface with a strong genetic predisposition. Below it, the aorta often presents an irregular, calcified, arteriosclerotic profile, influenced by traditional risk factors (45). Atherosclerosis and hypertension are key factors in descending thoracic aortic aneurysm and AAA development (46, 47). Conversely, ascending thoracic aortic aneurysm and dissection are frequently linked to genetic factors like Marfan's syndrome (45), as well as Ehlers-Danlos syndrome, Loeys-Dietz syndrome, bicuspid aortic valve, and familial AD (48). Given these distinctions, it's crucial to recognize that aortic diseases are heterogeneous. Thoracic, descending, and abdominal aortic diseases each have unique physiological and genetic characteristics. Therefore, applying mechanisms of T2DM's influence on abdominal aortic lesions to AD may not be accurate. A nuanced, comprehensive approach is needed to understand the complex interactions and causal relationships in these varied aortic conditions.

Previous clinical and basic research suggest that diabetes may affect the occurrence and progression of AD through several mechanisms. T2DM may modify the aortic wall's biological structure, as hyperglycemia promotes collagen cross-linking in the aortic media, enhancing resistance to proteolysis and reducing matrix metalloproteinases (MMPs) secretion, key factors in aortic aneurysm development. Additionally, T2DM's inhibition of plasmin, an MMP activator, could lessen aortic wall degradation (49), aligning with observations of thicker aortic walls in diabetics (46). However, some studies report increased MMP activity, particularly MMP-1, MMP-2, and MMP-9, with higher blood glucose levels, which could counteract any protective effect of diabetes against AD (50). Advanced glycation end products (AGEs) accumulation, another consequence of high glucose levels, leads to cross-linking in the extracellular matrix, stiffening the aortic wall, which could be protective against AD (9, 51–53). On the other hand, increased AGEs and their receptors (RAGEs) may promote inflammation, contributing to aortic disease (54). In complex pathophysiological environments such as lesioned aortic walls, not all data can be expected to be consistently concordant.

Beyond diabetes-induced hyperglycemia, other factors may affect AD occurrence. Diabetes medications, like thiazolidinediones and metformin, could protect against AD due to their role in reducing MMP expression in the aortic wall (55, 56). Insulin resistance (IR) is another potential risk factor. Clinical studies, mouse models, and cellular research indicate that IR triggers vascular smooth muscle cells to switch from contractile to synthetic phenotypes, contributing to AD development (57). In older adults without diabetes, IR has been linked to aortic stiffness, a condition that might alter hemodynamics and promote AD (58). Furthermore, diabetic patients often are subject to heightened attention to blood pressure management and other secondary preventive strategies. Each of the above factors has the potential to influence the occurrence of AD independently.

We note that Weizong Zhang et al. conducted a MR study on the association between T2DM and AD (59). This study utilized 26 diabetes datasets to confirm that there is no causal relationship between the genetic susceptibility to T2DM and AD. Compared to the aforementioned study, our research has the following advantages: Given the significant racial differences in genetic characteristics, our data set consists exclusively of individuals of European ancestry, with a sample size larger than that in the previous study. For outcome data, we validated our findings using the UK Biobank data of non-Finnish individuals and used coronary heart disease data from CARDIoGRAMplusC4D as a positive control to verify the reliability of our instrumental variables. Furthermore, to enhance the comprehensiveness of our analysis, we also included important biomarkers such as FG, FI, and HbA_1c_ as exposures to analyze their impact on aortic dissection. It is important to note that even in MR studies, it is challenging to avoid confounding factors. Therefore, we analyzed 29 variables, including hypertension, which could introduce confounding bias, and conducted multivariable Mendelian randomization to exclude potential interferences. However, the study also faces limitations. Privacy concerns and the lack of detailed data in public GWAS databases impeded our ability to differentiate AD patients by gender, type, or syndromic status. Additionally, the relative rarity of AD limits the categorization of these factors in public GWAS databases, impacting our analysis. A key limitation is our reliance on genetic data, posing challenges in discerning specific impacts of late-onset exposures and their compensatory effects. Assessing the impact of diabetes metabolic products and hypoglycemic treatment on AD is difficult. While MR reduces confounding effects, unidentified physiological pathways in AD's complex pathology could introduce biases. The robustness of MR depends on large sample sizes and comprehensive genetic data, yet the infrequency of AD in the general population limits data availability. Moreover, as our study is based on a European demographic, the findings might not be universally applicable.

## 5 Conclusion

In summary, this MR study indicates that the genetic susceptibility to T2DM does not confer a protective effect against AD. These findings contrast with previous observational evidences, which suggest a protective effect of T2DM. This discrepancy underscores the need for further comprehensive research, particularly focused on exploring the potential preventative role of common T2DM treatment modalities in AD. Unraveling the intricate relationships between T2DM and AD is a crucial step that not only enriches our understanding of AD's complex pathobiology but also paves the way for innovative therapeutic interventions for this elusive disease.

## Data Availability

The datasets presented in this study can be found in online repositories. The names of the repository/repositories and accession number(s) can be found in the article/**supplementary Material**.
