# Supplementary material for: Genetic predisposition to type 2 diabetes mellitus and aortic dissection: a Mendelian randomisation study
Source: Front Cardiovasc Med. 2024 Jul 22;11:1382702. doi: 10.3389/fcvm.2024.1382702 (PMC11298347; doi:10.3389/fcvm.2024.1382702)
Supplement: Supplementary file 2 [file Datasheet2.docx]

**Supplementary Figure 1.** Scatter plots of Mendelian randomization (MR) analyses for type 2 diabetes mellitus (T2DM) with aortic dissection (AD) of primitive group.(A) Funnel plots of MR analyses for T2DM with AD of primitive group.(B) Scatter plots of MR analyses for T2DM with AD of adjustment group.(C) Funnel plots of MR analyses for T2DM with AD of adjustment group.(D) Scatter plots of MR analyses for T2DM with AD of validation set.(E) Funnel plots of MR analyses for T2DM with AD of validation set.(F)

**Supplementary Figure 2.** Leave one out analysis of Mendelian randomization (MR) analyses for type 2 diabetes mellitus (T2DM) with aortic dissection (AD) of primitive group.(A) Forest plots of MR analyses for T2DM with AD of primitive group.(B) Leave one out analysis of MR analyses for T2DM with AD of adjustment group.(C) Forest plots of MR analyses for T2DM with AD of adjustment group.(D) Leave one out analysis of MR analyses for T2DM with AD of validation set.(E) Forest plots of MR analyses for T2DM with AD of validation set.(F)

**Supplementary Figure 3.** Scatter plots of Mendelian randomization (MR) analyses for fasting glucose (FG) with aortic dissection (AD) of primitive group.(A) Funnel plots of MR analyses for FG with AD of primitive group.(B) Scatter plots of MR analyses for FG with AD of adjustment group.(C) Funnel plots of MR analyses for FG with AD of adjustment group.(D)

**Supplementary Figure 4.** Leave one out analysis of Mendelian randomization (MR) analyses for fasting glucose (FG) with aortic dissection (AD) of primitive group.(A) Forest plots of MR analyses for FG with AD of primitive group.(B) Leave one out analysis of MR analyses for FG with AD of adjustment group.(C) Forest plots of MR analyses for FG with AD of adjustment group.(D)

**Supplementary Figure 5.** Scatter plots of Mendelian randomization (MR) analyses for fasting insulin (FI) with aortic dissection (AD) of primitive group.(A) Funnel plots of MR analyses for FI with AD of primitive group.(B) Scatter plots of MR analyses for FI with AD of adjustment group.(C) Funnel plots of MR analyses for FI with AD of adjustment group.(D)

**Supplementary Figure 6.** Leave one out analysis of Mendelian randomization (MR) analyses for fasting insulin (FI) with aortic dissection (AD) of primitive group.(A) Forest plots of MR analyses for FI with AD of primitive group.(B) Leave one out analysis of MR analyses for FI with AD of adjustment group.(C) Forest plots of MR analyses for FI with AD of adjustment group.(D)

**Supplementary Figure 7.** Scatter plots of Mendelian randomization (MR) analyses for hemoglobin A_1c_ (HbA_Ic_) with aortic dissection (AD) of primitive group.(A) Funnel plots of MR analyses for HbA_Ic_ with AD of primitive group.(B) Scatter plots of MR analyses for HbA_Ic_ with AD of adjustment group.(C) Funnel plots of MR analyses for HbA_Ic_ with AD of adjustment group.(D)

**Supplementary Figure 8.** Leave one out analysis of Mendelian randomization (MR) analyses for hemoglobin A_1c_ (HbA_Ic_) with aortic dissection (AD) of primitive group.(A) Forest plots of MR analyses for HbA_Ic_ with AD of primitive group.(B) Leave one out analysis of MR analyses for HbA_Ic_ with AD of adjustment group.(C) Forest plots of MR analyses for HbA_Ic_ with AD of adjustment group.(D)

**Supplementary Figure 9.** The association between exposures and coronary artery disease(CAD) using the inverse variance weighted method.(A) Scatter plots of Mendelian randomization (MR) analyses for type 2 diabetes mellitus (T2DM) with CAD.(B) Funnel plots of MR analyses for T2DM with CAD.(C) OR = odds ratio; CI = confidence interval; P = probability value_._

**Supplementary Figure 10.** Scatter plots of Mendelian randomization (MR) analyses for fasting glucose (FG) with coronary artery disease(CAD).(A) Funnel plots of MR analyses for FG with CAD.(B) Scatter plots of MR analyses for fasting insulin (FI) with CAD.(C) Funnel plots of MR analyses for FI with CAD.(D) Scatter plots of MR analyses for hemoglobin A_1c_ (HbA_Ic_) with CAD.(E) Funnel plots of MR analyses for HbA_Ic_ with CAD.(F)
